# Suprahyoid Muscle Activity in Patients with Chagasic Megaesophagus

**DOI:** 10.1038/s41598-019-55402-5

**Published:** 2019-12-11

**Authors:** Aretuza Zaupa Gasparim El Gharib, Giédre Berretin-Felix, Roberto Oliveira Dantas, Diogo Francisco Rossoni, Max Jean de Ornelas Toledo

**Affiliations:** 1 Postgraduate Program in Health Sciences, Health Sciences Center, State University of Maringá, Maringá, PR Brazil; 2 Department of Speech-Language Pathology and Audiology, Bauru School of Dentistry, University of São Paulo, Bauru, SP Brazil; 3 Department of Medicine, Ribeirão Preto Medical School, University of São Paulo, Ribeirão Preto, SP Brazil; 4 Department of Statistics, State University of Maringá, Maringá, PR Brazil; 5 Department of Basic Health Sciences, State University of Maringá, Maringá, PR Brazil

**Keywords:** Dysphagia, Preclinical research

## Abstract

The objective of this investigation was to evaluate the activity of the suprahyoid musculature during swallowing and to correlate the findings with the degree of megaesophagus, oral and pharyngeal videofluoroscopy and esophageal manometry in patients with achalasia caused by Chagas’ disease. Twenty-nine patients with positive serology for *Trypanosoma cruzi* and dysphagia (Chagas’ disease group) and 29 individuals matched by sex and age (control group) participated in the study. Surface electromyography of the suprahyoid musculature and videofluoroscopy during swallowing of paste and liquid consistencies were performed. Canonical correlation analysis of the MANOVA test results showed that the Chagas’ disease group had lower electromyographic activity when compared with controls. Overlapping circles of radiological findings were found for megaesophagus. The Spearman test showed a positive correlation between the electromyographic activity in the maximum voluntary isometric contraction and the time of pharyngeal transit for both liquid (*p* = 0.014) and paste (*p* = 0.047). The logistic regression test showed no association between electromyographic activity of the suprahyoid muscles and esophageal manometry results (p > 0.05). In conclusion, individuals with chagasic megaesophagus have reduced electromyographic activity of the suprahyoid muscles during swallowing, in addition to a greater recruitment of the suprahyoid musculature with increased pharyngeal transit time.

## Introduction

Chagas’ disease (CD) or American trypanosomiasis is a potentially fatal infectious tropical disease of high morbidity, caused by the parasitic protozoan *Trypanosomacruzi*. The number of people infected in the endemic area of Latin America, in addition to the hundreds of thousands in non-endemic areas in North America, Europe, Australia and Japan due to migration, is estimated to be about 6 million to 7 million people around the world^1^.

The disease mainly affects the heart and gastrointestinal tract, leading to dilated cardiomyopathy, megacolon and megaesophagus^2^. Although the digestive form of the disease is common in central Brazil and Chile, it is practically non-existent in Amazonia, Colombia, Venezuela and Central America^3,4^. In the State of Paraná, Southern Brazil, 14% and 20% of the patients present the digestive and cardiodigestive forms of CD, respectively^5^, with dysphagia as one of the most frequent symptoms (26% of patients).

In chagasicmegaesophagus, destruction of the myenteric plexus caused by *T. cruzi*^6^ is responsible for functional changes, such as hypocontractility, motor dyskinesia and incomplete or absent relaxation of the lower esophageal sphincter (LES)^7^, the classical presentation of esophageal achalasia. Esophageal symptoms, altered motility and increase in esophageal diameter is seen in 7% to 10% of infected subjects^8^. However,oropharyngealmanifestations may occur over the course of the disease^9^.

In oral and pharyngeal phases of swallowing in patients with CD an increase in oral residues, longer pharyngeal clearance for paste bolus^10^, longer pharyngeal clearance and upper esophageal transit^11^, longer pharyngeal transit andlonger opening of the upper esophageal sphincter (UES)^9^ were observed.

Assessment of esophageal function in CD is performed by radiological examination^12,13^ and conventional^8^ or high-resolution manometry^14^. So far, studies of muscle electrical activity by surface electromyography (EMG) in patients with CD have not been performed. Thistechnique has beenused as anaid in differentialdiagnosisandmonitoringof possible muscular disorders^15^ and contributed to the assessment of swallowing dysfunction.

The suprahyoidmuscles are consideredstrategic in swallowing, as theyparticipate in reflex motor mechanisms^16^, mandibular loweringandstabilization^17^, and elevationandanteriorizationofthehyoidbone^18^, andhaveattachmentstothetongue musculature^16^. The activity of these muscles is modified by bolus consistency and actions of the muscles involved^19^.

Our hypothesis was that in achalasia caused by CD suprahyoid muscle activity is altered during swallowing, which may be associated with changes in pharyngeal transit and esophageal motility.To test this hypothesis, the activity of the swallowing-relatedmuscles was evaluated using surface electromyography (EMG) and correlated with megaesophagus grade, videofluoroscopic evaluation of swallowing, and esophageal manometry.

## Materials and Methods

### Ethical aspects

This study and all methods were performed in accordance with the relevant guidelines and regulations. It was approved by the Committee of Ethics in Research with Human Beings of the State University of Maringá (UEM) (Certificate of Submission for Approval of Ethics - number 45350415.0.0000.0104). All patients who participated in the study signed the informed consent form.

### Participants

Participants were recruited from the UEM Laboratory of CD and evaluated during the period from December 2016 to May 2017. Inclusion criteria for the CD group were: reactive serologic test for *T. cruzi* infection, megaesophagus and complaints of dysphagia, and exclusion criteria were: negative serology for *T. cruzi* infection, absence of dysphagia, previous neurological or oncological diseases, and refusal to perform diagnostic evaluations. The control group (CG) consisted of individuals with negative serology for *T. cruzi*, with no history of head and neck cancer, without digestive diseases, neurological diseases, or dysphagia and was matched by sex and age with the CD group.

Each patient of the CD group was assessed by serology, esophageal manometry, esophageal radiology, suprahyoid EMG and oral-pharyngeal videofluoroscopy, and each participant ofthe CG underwent serology and EMG evaluations.

### Serological testing

Anti-*T. cruzi* IgG antibodies were detected by chemiluminescence^20^.

### Esophagography

Megaesophagus grade in patients with CD was defined radiologically. Anteroposterior radiographs were taken considering four degrees of megaesophagus^12^: Grade I - small amount of barium retention visible in the esophageal lumen, without increase in esophageal diameter; Grade II - moderate esophageal dilation, significant retention of contrast and presence of tertiary waves; Grade III - hypotonic esophagus with significantly increased diameter, little contractile activity of the wall; and Grade IV – cases of dolichomegaesophagus, esophagus with large retention capacity, elongated, folding over the diaphragmatic dome.

### Surface electromyography

Surface EMG was performed using a four-channel Miotool (Miotec®) device. The channels were calibrated at 500 microvolts (μV) with bandpass filters of 20–500 Hz, and notch filters of 60 Hz. An amplification factor of 2000 was used for the EMG signals. The software used for EMG data processing was Miotec® Miograph 2.0, which performs online acquisition, storage and processing of the signals. Electromyographic activity of the right (RSH) and left (LSH) suprahyoid muscles were evaluated.

The ground electrode was placed on the olecranon of the right arm to minimize interference from external noise^21^. The following tests were performed: (1) maximum voluntary isometric contraction (MVIC) of the suprahyoid muscles by pressing the tongue against the palate^22,23^ for three seconds with mouth ajar; (2) swallowing liquid (SL) (5 mL in a single swallow); liquid bolus was prepared with 2.5 mL of water at room temperature plus 2.5 mL of liquid barium (Opti-Bar); and (3) swallowing paste (SP) (5 mL in a single swallow). Paste bolus was prepared with 3.6 g of ThickenUp Clear food thickener, 50 mL of water and 50 mL of liquid barium stirred until complete dissolution. The liquid bolus was classified as level 0 (thin liquid) and the paste bolus as level 3 (moderately thick) according to the International Dysphagia Diet Standardisation Initiative (IDDSI) classification^24^. The eletromyographic activity was expressed as root mean square (RMS) of the amplitude (μV). Three swallows were performed, with an interval of 10 seconds between them, and the mean of three records was used for analysis.

### Videofluoroscopic swallowing study

Videofluoroscopic swallowing study of CD patients was performed using a fluoroscopy device equipped with a closed-circuit television and X-ray image intensifier (Toshiba rotating anode tube, 500 mA); image was displayed at 30 frames per second.

The videofluoroscopic evaluation included the oral, pharyngeal and esophageal (proximal esophagus) phases of swallowing^25^. Two swallows of 5 mL (liquid and paste) were investigated at lateral and frontal positions. Liquid and paste boluses were placed in each participant’s mouth using a 10 mL syringe.

Videofluoroscopic swallowing examination was performed with the individuals in standing position, who were asked to move their bodies from lateral to frontal position as appropiate. The anatomical boundaries visualized in the videofluoroscopic field were: upper and lower limits from the oral cavity to the esophagus, with the lips defined as the anterior border and the pharyngeal wall as the posterior border, and the upper nasopharynx and the cervical esophagus defined as the upper and the lower limits^25^.

Oral transit time (OTT) and pharyngeal transit time (PTT) were calculated by image analysis, with the aid of the tracer marker of the Kinovea software - 0.8.15 (Copyright © 2006–2011 - Joan Charmant and Contrib.), which enables a frame-by-frame analysis every 3 milliseconds. The OTT was defined as the interval between the first frame showing movement of the food bolus to the first frame in which the head of the bolus reached the lower edge of the mandible. PTT was measured from the first video frame in which the head of the bolus reached the lower edge of the mandible to the first frame where the tail of the bolus passed through the upper esophageal sphincter^26^.

### Esophageal manometry

Esophageal manometry was performed in the CD group using a 2.3 mm diameter eight-channel polyvinyl catheter 4 distal (radial) channels and 4 spirally aligned channels spaced at 5-cm intervals, perfused continuously with distilled water. Pressures were transmitted to external pressure transducers, to which the channels were connected (Dynamed, São Paulo, Brazil), recorded by a polygraph and analyzed using the Gastro-Master 7 software (Dynamed, São Paulo, Brazil).

The catheter was introduced through the nostril after a 12-hour fast, under local anesthesia with 2% lidocaine gel, with the patient seated and head flexed. As the catheter entered the esophagus, the patient was asked to lie down. Through the four distal channels, mean intragastric pressure was measured. The resting pressure of the LES was measured by the station pull-through method, taking the intragastric pressure as a reference and the maximum expiratory point as the result. With this same method, we determined the location and extent of the LES. The relaxation of the LES was evaluated by measuring the pressure during three swallows of 5 mL of doubly distilled water at room temperature, with a minimum interval of 30 seconds between them^27^.

For recordings contractions of the esophageal body, the most distal channel was located 3 cm above the upper border of the LES, and the other three channels located 5 cm apart. The subjects performed 10 swallows of 5 mL of liquid bolus (water). The contraction amplitude of the esophageal body, measured with patients breathing normally, was defined as the difference between intra-esophageal pressure between swallows and the peak of the contraction wave. Its duration was measured from the beginning of the contraction until return to pre-contraction values. Measurement of the resting upper esophageal sphincter (UES) was performed with six channels, with the proximal two placed in the pharynx and the distal ones in the UES.

### Statistical analysis

The sample was stratified (e.g. patients with megaesophagus III and IV) by qualitative and quantitative variables for statistical analysis. Univariate (ANOVA) and multivariate analysis (MANOVA) were performed to determine differences in EMG values between CD and control groups. Canonical correlation analysis was performed following MANOVA to stablish correlations between the study variables.

Mean eletromyographic activity values RSH and LSH muscles were used for correlation analysis, since there was no statistically significant difference between the sides. Correlations between oral and pharyngeal transit times were determined by the Spearman test.

For esophageal manometry results, mean values of RSH and LSH muscles were first used, and then classified between 0 and 1 (within or outside the normal range of each variable) and the Logistic Regression test was applied. This test was used to compare the EMG and esophageal manometry results at 0.05 significance. All analyses were done in R (R Core Team, 2017) software.

## Results

In the study period, 34 patients were evaluated, of which five were excluded. The reasons for exclusion were: two (40%) with non-reactive serology for *T. cruzi*, two (40%) with absence of megaesophagus on radiography, one (20%) for refusal to perform clinical diagnostic tests. The CD group consisted of 29 patients aged 46 years to 73 years (median 63 years), 21 (72.4%) of them were women. Positive chemiluminescence response to anti-*T. cruzi* antibodies was seen in all patients. Twelve (41.4%) had megaesophagus grade I, nine (31.0%) grade II and eight (27.4%) grades III/IV. The CG consisted of 29 subjects aged 48 to 73 years, median 62 years, of whom 21 (72.4%) were women.

The electromyographic activities of the CG and CD groups underwent univariate analysis by using the mean and standard deviation values of the RSH and LSH muscles separately, and after, subjected to multivariate analysis, applying the ANOVA test at a 0.05 significance level to verify if there was a difference between CG and CD (Table 1).

**Table 1:** Electromyographic activity of the right and left suprahyoid muscles, expressed as root mean square of the amplitude (microvolts) in subjects with Chagas’ disease and control group.

Variables of EMG	Control	Chagas’ disease	*p*-value*
MVIC – RSH	56.8 (19.7)	33.3 (23.4)	0.002
MVIC – LSH	57.6 (21.1)	34.9 (23.6)	0.003
SL – RSH	48.9 (12.4)	29.7 (13.9)	<0.001
SL – LSH	49.4 (13.5)	32.0 (15.1)	0.000
SP – RSH	53.1 (19.4)	31.1 (11.4)	<0.001
SP – LSH	54.2 (22.2)	33.5 (13.4)	0.000

MVIC: maximum voluntary isometric contraction; SL: swallowing liquid; SP: swallowing paste; RSH: right suprahyoid muscle; LSH: left suprahyoid muscle; *p values < 0.005. Data presented as mean (standard deviation).

**Figure 1:**
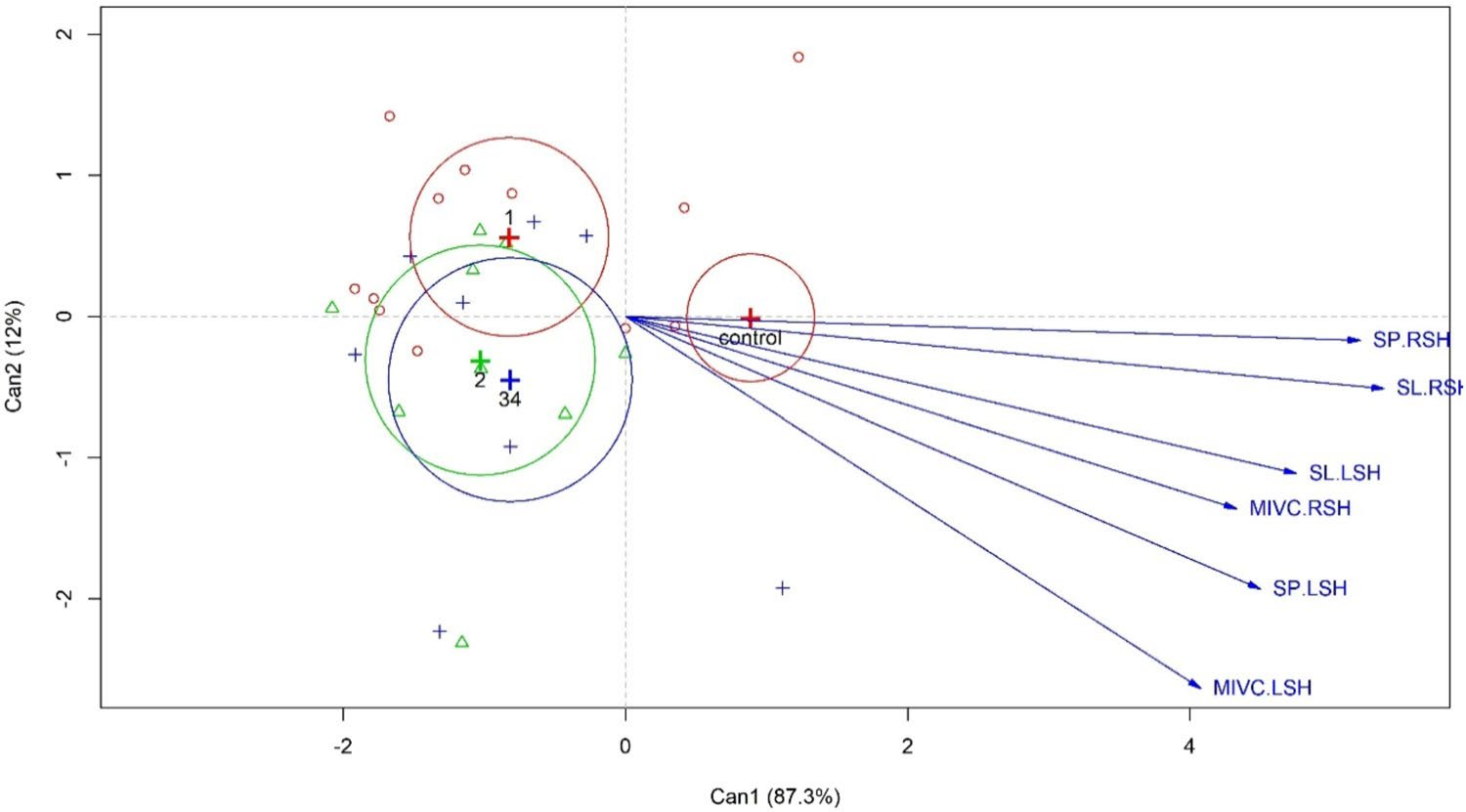
Canonical correlation analysis of the electromyographic activities of the supra-hyoid muscles in patients with Chagas’ disease and controls. MVIC: maximum voluntary isometric contraction; RSH: right suprahyoid muscles; LSH: left suprahyoid muscles; SL: swallowing liquid; SP: swallowing paste; degrees of megaesophagus 1 (I), 2 (II), 34 (III/IV).

After confirming the significance (by p-value from Table 1), the canonical correlation analysis was performed from the results of the MANOVA test at a 0.05 significance level, which showed the differences between the groups and correlated the study variables (Fig. 1). A lower electromyographic activity was observed in the CD group compared with the CG. We observed an overlapping of the circles of megaesophagus grades, mainly in grades II and III/IV in canonical analysis. However, a slight increase in muscle recruitment was observed in grade I compared with the other grades, suggesting that a higher degree of megaesophagus may be associated with a decrease in the electromyographic activity.

Regarding EMG values, there was a positive correlation of RSH muscle activity with elecromiographic activities for both PS and LS. In contrast, EMG activity was significantly lower for the LSH muscles compared with the RSH muscle.

Mean and standard deviation of the oral and pharyngeal transit times for LS and PS analyzed by videofluoroscopy, are presented in Table 2.

**Table 2:** Oral and pharyngeal transit times (in seconds) analyzed by videofluoroscopy swallowing study in patients with Chagas’disease (n = 29).

Swallowing of liquid		Swallowing of paste
OTT	0.67 (0.45)	1.27 (1.21)
PTT	0.42 (0.43)	0.51 (0.71)

OTT: oral transit time; PTT: pharyngeal transit time. Data presented as mean (standard deviation).

Table 3 shows the results of the Spearman correlation analysis of oral and pharyngeal transit times by EMG in CD group. The variable PTT for liquid and paste showed a positive correlation with the electromyographic activity of the MVIC; that is to say, the longer the PTT, the greater the electromyographic activity of the suprahyoid muscles.

**Table 3:** Correlation (Spearman test) between oral transit time (OTT), pharyngeal transit time (PTT) and surface electromyography (EMG) in patients with Chagas’ disease (n = 29).

EMG	OTT – SL		OTT – SP		PTT–SL		PTT – SP	
	r	*p* value	r	*p* value	r	*p* value	r	*p* value
MVIC	0.087	0.650	−0.082	0.669	0.450	0.014*	0.370	0.047*
SL	−0.323	0.086	−0.219	0.252	0.172	0.369	0.185	0.335
SP	−0.033	0.864	−0.239	0.210	−0.145	0.450	0.006	0.971

OTT: oral transit time; PTT: pharyngeal transit time; MVIC: maximal voluntary isometric contraction; SL: swallowing liquid; SP: swallowing paste; *p values < 0.05.

Esophageal manometry results are presented in Table 4. The logistic regression test did not show any correlation between electromyographic and esophageal manometric results in the group of patients with CD (Table 5).

**Table 4:** Esophageal manometry results in patients with Chagas’ disease (n = 29).

		Mean (SD)
LES	Resting Pressure (10–35 mmHg)	11.3 (10.7)
Esophageal body	Distal Amplitude (59–139 mmHg)	35.0 (29.2)
	Duration (3–4.8 s)	7.5 (2.7)
UES	Resting Pressure (40–130 mmHg)	45.9 (15.4)

SD: standard deviation; LES: lower esophageal sphincter; UES: upper esophageal sphincter. Data presented as mean (standard deviation).

**Table 5:** Correlation (logistic regression test) between the variables of the esophageal manometry and surface electromyography (EMG) values in patients with Chagas’ disease (n = 29).

EMG	LES	Esophageal body		UES
	Resting Pressure	Distal Amplitude	Distal Duration	Resting Pressure
	*p* value	*p* value	*p* value	*p* value
MVIC	0.555	0.786	0.326	0.872
SL	0.230	0.547	0.408	0.332
SP	0.415	0.572	0.294	0.150

LES: lower esophageal sphincter; UES: upper esophageal sphincter; MVIC: maximum voluntary isometric contraction; SL: swallowing liquid; SP: swallowing paste.

## Discussion

In a previous study carried out by our group^5^, a total of 345 individuals with positive serology for *T. cruzi* infection were identified. Of them, 270 participated in the study and indeterminate (30%), cardiac (36%), cardiodigestive (20%) and digestive (14%) forms were found. That is, 34% (92 patients) presented digestive or cardiodigestive form, and 25.5% (89 patients) complained of dysphagia. In the present study, from the sample universe of 345 patients with CD, we evaluated 34/89 with dysphagia, ie, 38.2% of them.

The screening test for *T. cruzi* antibodies used in this study, chemiluminescence, was selected for its high diagnostic performance for CD. In the present study, the test showed a high sensitivity (100%), confirming previous data^28^.

EMG has been used to assess the activity of swallowing muscles, both in people with normal swallowing and in those with swallowing disorders^29^. When studying the electromyographic activity of individuals with and without CD, we observed that patients with CD had lower activity than those without CD. This can be explained by the decrease in muscle recruitment of the suprahyoid muscles in patients with chagasic megaesophagus and with complaints of dysphagia, a novel finding of this study.

The biomechanics of swallowing are directly linked to contraction of the suprahyoid muscles. This contraction promotes the elevation and stabilization of the laryngeal complex during swallowing, protecting the lower airway from saliva and/or food entry^30^. Analysis of the supra-hyoid musculature by EMG has usually included, the end of the oral phase, the pharyngeal phase and the beginning of the esophageal phase^16^. In the present study, we found that recruitment of the supra-hyoid muscles is affected in CD patients when compared with healthy individuals, indicating disturbances in muscular activity during the pharyngeal phase of swallowing, and consequently altered pharyngo-esophageal transit.

One limitation of this study was the proof of maximum voluntary isometric contraction (MVIC) of the suprahyoid muscles by pressing the tongue against the palate^22,23^ for three seconds with the mouth ajar. Further studies are needed to confirm the relationship between electromyographic activity and suprahyoid muscle contraction.

It is important to consider that the amplitude of the electrical activity, recorded by EMG in this study, is one of the important factors in the relationship between muscle recruitment and electrical activity^29^. However, amplitude variations in EMG are not only due to biological causes, but also due to technical factors such as skin/electrode impedance, anatomical location of recording electrodes, differences in muscle size between individuals, and temperature^15^.

In the canonical analysis of chagasic patients, there was an overlap of the circles, showing that there are no differences in the electromyographic activities in megaesophagus grades II and III/IV. There was, however, a slightly greater recruitment of muscles in grade I patients, suggesting not only an impairment in supra-hyoid muscle function but also a compensatory mechanism in more advanced degree of megaesophagus, which could contribute to esophageal dysphagia. We did not find any studies on EMG in individuals with CD, which makes it difficult to compare the results.

PTT is defined as the interval from the first video frame in which the first edge of the bolus reaches the posterior mandible to the first frame where the tail of the bolus passes through the UES^31^. The PTT for liquid and paste showed a positive correlation with the electromyographic activity obtained during the test of MVIC i.e., the longer the PTT, the higher the number of motor units activated for the suprahyoid muscles. Ineffective food clearance in the pharynx may be a consequence of incomplete opening of the UES, decreased contraction pressure of the pharyngeal and/or tongue musculature^32,33^, requiring greater muscle recruitment in swallowing, which would justify the correlation found. This suggests that the organism recruits more motor units so as to compensate the long PTT, thus, providing safe swallowing, since, according to the literature^34^, the long transit by the pharynx may be associated with greater aspiration possibilities upon swallowing. The confirmation of this hypothesis needs further investigation.

Dysmotility associated with advanced degree of megaesophagus causes resistance to the flow at the entry of the bolus into the esophagus^33^. Slower PTT may be a consequence of resistance to pharyngeal peristalsis itself^9^ and intra-bolus pressure in the hypopharynx augments with increasing bolus consistency^35^. The slow transit by the pharynx may derive from the resistance of bolus entry into the esophagus. Hence, the lower EMG activity in patients presented with CD, in relation to the controls, verified in the present study, may result from lower pressure needed to force the entry into the esophagus, thus, worsening the esophageal dysphagia presented by the patient.

It is known that patients with CD have different degrees of motor impairment throughout the digestive tract^7,8^. Regarding the comparison of EMG and esophageal manometry test results, we did not find any statistically significant difference for any of the studied variables. One possible hypothesis to explain such results is that the difficult in the transit by the esophagus is not dependant on the degree of the megaesophagus and, therefore, causes the same response in the muscular function observed by the EMG. Patients with degree I radiological condition, with no dilatation, may show greater difficulty in the transit by the proximal part of the esophagus than those with dilatation. On the other hand, the difficulty in the megaesophagus is in the distal part, in the transition between the esophagus and the stomach. The literature on this issue is scarce and further studies should be carried out, so as to give support to this hypothesis.

This study represents the first comparison of EMG activity of the suprahyoid musculature during swallowing between individuals with chagasic megaesophagus and those without it, correlating EMG to videofluoroscopy and manometry tests. It was found that the longer the PTT, the greater the activity of suprahyoid muscles, even if recruitment in the CD group is lower than that of the controls, which may be seen as an attempt to correct or compensate the impairment.

It is an important contribution to the understanding of oropharyngeal aspects of swallowing in this population, since, although changes in esophageal motility are the main cause of dysphagia in CD, all stages of swallowing may be impaired^7–9^. Understanding the involvement of the suprahyoid muscle function during swallowing in patients with chagasic megaesophagus opens new perspectives for specific speech-language intervention for this population.

One limitation of this study is that we did not perform videofluoroscopic or manometric test in CG. However, previous manometric studies have well described changes in esophageal motility in CD patients^7,8,14^. Also, studies involving videofluoroscopic swallowing examination have shown that pharyngeal transit duration, sometimes named pharyngeal clearance, is longer in this population^9,11^.

## Conclusion

CD patients have reduced electromyographic activity of the suprahyoid muscles during swallowing, which may be more pronounced in those with higher degree of megaesophagus. Electromyographic activity in MVIC was positively correlated with the PTT. No correlation was found between manometry and EMG values. These results suggest the involvement of the suprahyoid muscle function during swallowing in patients with chagasic megaesophagus.
